# Electronic Integrated Management of Childhood Illness (eIMCI): a randomized controlled trial to evaluate an electronic clinical decision-making support system for management of sick children in primary health care facilities in South Africa

**DOI:** 10.1186/s12913-024-10547-6

**Published:** 2024-02-08

**Authors:** C. Horwood, L. Haskins, S. Mapumulo, C. Connolly, S. Luthuli, C. Jensen, D. Pansegrouw, N. McKerrow

**Affiliations:** 1 Centre for Rural Health, School of Nursing and Public Health, University of KwaZulu-Natal, Durban, South Africa; 2 Health Systems Strengthening Unit, Health Systems Trust, Durban, South Africa; 3 KwaZulu-Natal Department of Health, Ilembe District, Stanger, South Africa; 4 KwaZulu-Natal Department of Health, Paediatrics and Child Health, Pietermaritzburg, South Africa; 5 Department of Paediatrics and Child Health, University of Cape Town, Cape Town, South Africa; 6 Department of Paediatrics and Child Health, University of KwaZulu-Natal, Durban, South Africa

**Keywords:** Electronic decision-making support system, Integrated management of childhood illness, Child health, IMCI, South Africa, Africa

## Abstract

**Background:**

Electronic clinical decision-making support systems (eCDSS) aim to assist clinicians making complex patient management decisions and improve adherence to evidence-based guidelines. Integrated management of Childhood Illness (IMCI) provides guidelines for management of sick children attending primary health care clinics and is widely implemented globally. An electronic version of IMCI (eIMCI) was developed in South Africa.

**Methods:**

We conducted a cluster randomized controlled trial comparing management of sick children with eIMCI to the management when using paper-based IMCI (pIMCI) in one district in KwaZulu-Natal. From 31 clinics in the district, 15 were randomly assigned to intervention (eIMCI) or control (pIMCI) groups. Computers were deployed in eIMCI clinics, and one IMCI trained nurse was randomly selected to participate from each clinic. eIMCI participants received a one-day computer training, and all participants received a similar three-day IMCI update and two mentoring visits. A quantitative survey was conducted among mothers and sick children attending participating clinics to assess the quality of care provided by IMCI practitioners. Sick child assessments by participants in eIMCI and pIMCI groups were compared to assessment by an IMCI expert.

**Results:**

Self-reported computer skills were poor among all nurse participants. IMCI knowledge was similar in both groups. Among 291 enrolled children: 152 were in the eIMCI group; 139 in the pIMCI group. The mean number of enrolled children was 9.7 per clinic (range 7-12). IMCI implementation was sub-optimal in both eIMCI and pIMCI groups. eIMCI consultations took longer than pIMCI consultations (median duration 28 minutes vs 25 minutes; *p* = 0.02). eIMCI participants were less likely than pIMCI participants to correctly classify children for presenting symptoms, but were more likely to correctly classify for screening conditions, particularly malnutrition. eIMCI participants were less likely to provide all required medications (124/152; 81.6% vs 126/139; 91.6%, *p*= 0.026), and more likely to prescribe unnecessary medication (48/152; 31.6% vs 20/139; 14.4%, *p* = 0.004) compared to pIMCI participants.

**Conclusions:**

Implementation of eIMCI failed to improve management of sick children, with poor IMCI implementation in both groups. Further research is needed to understand barriers to comprehensive implementation of both pIMCI and eIMCI. (349)

**Clinical trials registration:**

Clinicaltrials.gov ID: BFC157/19, August 2019.

## Background

Electronic clinical decision-making support systems (eCDSS) can be used to support clinicians as they make complex decisions about patient management while taking multiple factors into account [1]. eCDSSs are designed to be aligned with the consultation, guiding the user through the clinical process and providing guidance on patient management [1]. An eCDSS should be based on the latest evidence-based guidelines, and can be regularly and rapidly updated as guidelines change. Such tools have been shown to improve adherence to guidelines and improve quality of clinical decision-making in a number of settings [2, 3], and can also improve rational prescribing and effectively prevent medical errors [1, 4]. However, evidence is lacking for use of eCDSSs in low resource settings where the need for clinical support is greatest [4–6], but where information technology (IT) infrastructure and skills may be poor [7]. Nevertheless, the use of an eCDSS has potential to improve clinical care and patient outcomes in low resource settings where skilled health professionals and support and supervision are scarce but rates of preventable morbidity and mortality are high [4, 8].

The Integrated Management of Childhood Illness strategy (IMCI) was developed in response to the large number of global under-five deaths in low- and middle-income countries in the 1990’s [9]. IMCI provides evidence-based guidelines for management of sick children in primary health care (PHC) settings using a standardized algorithmic approach to guide IMCI practitioners step-by-step through the consultation [10]. IMCI has been implemented in more than 75 countries globally, including South Africa [11], and studies have shown that IMCI implementation can improve morbidity and mortality among under-five children [12, 13]. However, concerns have been raised about sustaining the quality of IMCI, and several studies have shown poor and fragmented IMCI implementation, with health workers omitting key components of the guidelines and missing opportunities to provide comprehensive care, or failing to use IMCI at all [14–16]. Reasons for poor implementation and non-adherence to IMCI guidelines are multiple and complex, and include frequent staff rotation, inadequate staffing and weak health systems, as well as poor motivation of health workers [17, 18]. Paper-based guidelines can be cumbersome in a busy clinical setting [4]. Further, ongoing support and supervision is crucial for successful IMCI implementation but has rarely been sustained adequately at scale [11, 19].

Thus, IMCI is an example of a well-established, widely implemented, evidence-based public health intervention where poor adherence to guidelines has limited effective implementation. Management of sick children is complex, children often present with multiple co-existing complaints, requiring health workers to assess a variety of signs and symptoms and integrate several different treatment algorithms. For all these reasons IMCI is particularly suitable for development of an eCDSS with the potential to help health practitioners correctly follow the relatively complex IMCI algorithm [20], improve adherence to IMCI guidelines, and improve prescribing practices for sick children [21]. A number of electronic tools to support IMCI have been developed and electronic IMCI has been shown to improve clinical management, improve adherence to the guidelines and reduce unnecessary prescriptions [4, 21, 22].

In South Africa (SA) IMCI guidelines have been adapted to the local clinical setting, including the addition of algorithms to screen for and manage HIV and TB in children [23]. Paper-based IMCI (pIMCI) has been the standard of care for sick children attending PHC clinics in SA for over two decades. An electronic version of the SA IMCI algorithm, known as eIMCI, has been developed and has been successfully piloted in PHC clinics in one district in KwaZulu-Natal (KZN) [24]. In this paper we report the findings of a cluster randomized controlled trial (RCT) to determine the effectiveness of eIMCI compared to pIMCI, and determine whether eIMCI can improve adherence to IMCI guidelines, and improve the assessment and management of sick children attending PHC clinics.

## Methods

We conducted a cluster RCT of eIMCI implementation using a parallel group design to compare the performance of eIMCI practitioners and pIMCI practitioners in assessing, managing and treating sick children attending participating clinics. The primary outcome was the proportion of sick children who received all medications indicated in the eIMCI and pIMCI groups. Secondary outcomes were the duration of the consultations and the proportion of children assessed and classified correctly in the two groups.

### Study site

The study was conducted in PHC clinics in one district in KZN, which was chosen in partnership with the KZN Department of Health, based on having a well-established IMCI programme. The district is predominantly rural, situated on the east coast of KZN comprising 4 sub-districts and 6 small municipal towns covering an area of 3300 square kilometers with a population of 657,000 [25]. Average annual household income is low (R14 600; USD 800) with high number (47.1%) of female headed households [26].

Clinic nurses in the district provide a range of routine services for children, and IMCI is the standard of care for management of sick children. There were one regional and three district hospitals, three community health centers and 31 PHC clinics in the district at the time of the study. All PHC clinics reported having at least one IMCI trained professional nurse at the clinic. Between 11-12000 deliveries are conducted annually, 18% of these are to teenage mothers. Immunization coverage for children under 1 year is 98.3% and Vitamin A coverage is 84% [27]. Preventable conditions such as diarrhoeal disease and lower respiratory infections are among the leading causes of death in children <5 years in the district [25].

### Description of the intervention

**Fig. 1:**
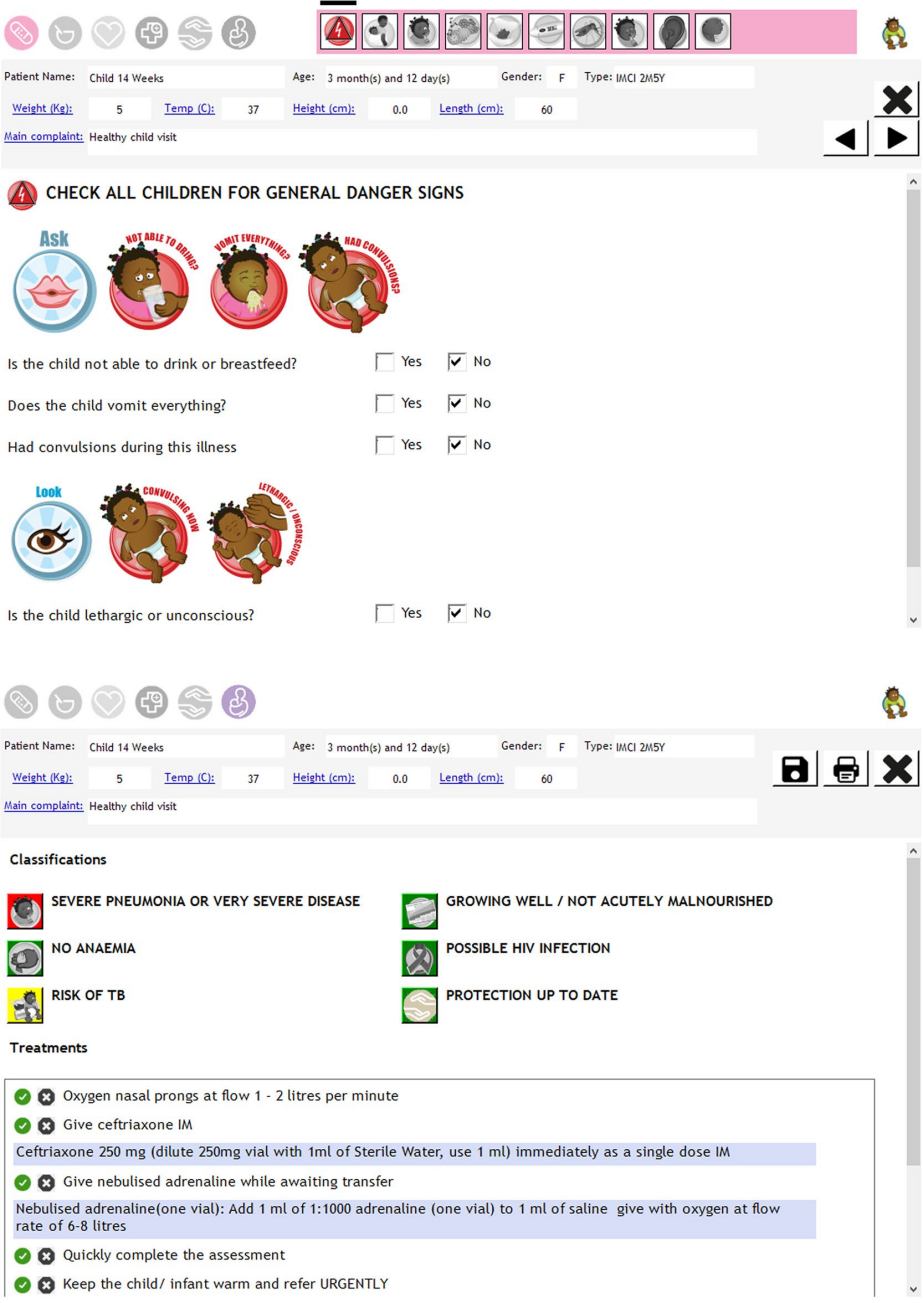
Examples of EIMCI user interface

eIMCI was developed as a component of the Virtual Electronic Medical Records system used by the KZN Department of Health (DoH). eIMCI was designed so that practitioners moved through the assessment in a similar way to when using pIMCI. Thus, eIMCI practitioners assessed each presenting symptom followed by nutrition and screening conditions (HIV and TB), entering their clinical findings as they moved through the assessment. Each part of the eIMCI assessment was mandatory, so practitioners could not proceed to the next section without completing the previous one. The exception was for presenting symptoms which were only assessed if marked as being present at the start of the consultation, for example if the practitioner failed to mark that fever or cough was present then these symptoms would be omitted from the assessment. Based on the information entered, eIMCI provided the classifications and recommended treatment for each child according to IMCI guidelines, and this could be printed and form part of the clinical records. Examples of the user interface are shown in Fig. 1.

Computers and printers were deployed in IMCI consulting rooms in all eIMCI clinics and the eIMCI application was installed on the computers before the start of the study. IT support was provided throughout the implementation period to minimise disruption to eIMCI for technical reasons.

Participants in both intervention and control groups received a similar IMCI update training and mentoring intervention at the start of the study. eIMCI participants received an additional one-day training on basic computer skills. Computer training was simple and based on the skills needed to use eIMCI, including logging into the computer, opening and closing eIMCI, mouse skills, entering patient information, saving and printing clinical findings. This was followed by a three-day IMCI update which included a series of case studies completed using eIMCI. pIMCI participants followed the same three-day training schedule including the same activities and case studies, but using paper-based IMCI. All participants received at least two mentoring visits by an IMCI trainer and were certified as IMCI competent before data collection started. Competency was based on ability to correctly assess and manage two sick children using IMCI, while being observed.

### Participants and sampling

**Fig. 2:**
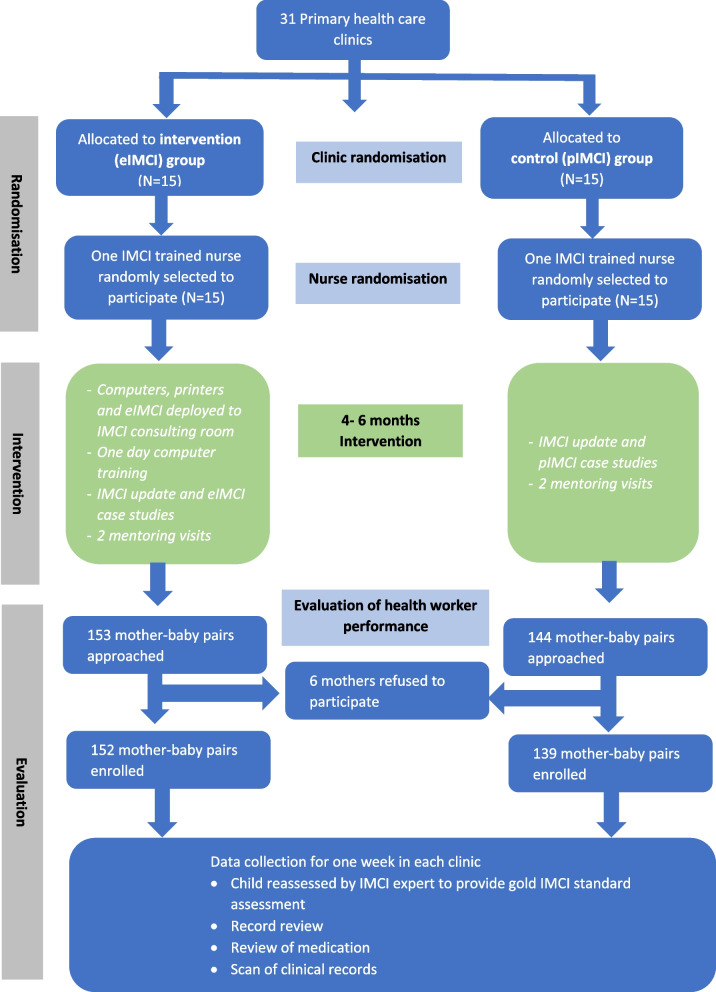
Study schema

The study was conducted in all selected clinics with selected IMCI trained nurses, using a two-stage sampling process as described below. All randomisation was conducted by a statistician using STATA v17 software. Four months after eIMCI training, performance of participants in both groups was assessed among selected mother-child pairs attending each participating clinic (Fig. 2).

#### Clinics

Participating clinics were systematically selected from a complete list of all 31 clinics in the district and 15 clinics were randomly allocated to the intervention group (eIMCI) and 15 to the control group (pIMCI).

#### IMCI practitioners

One IMCI trained nurse was randomly selected from among all IMCI trained nurses working in each clinic. Nurses who did not regularly provide sick child services or were not IMCI trained were excluded. Nurses were allocated to intervention or control groups based on the allocation of the clinic where they worked. The COVID-19 epidemic disrupted the start of the study and some nurses received study training shortly before the COVID-19 shutdown. The study restarted when COVID-19 restrictions were lifted and new IMCI trained nurses were recruited. Those IMCI trained nurses who had already received IMCI refresher training were excluded to ensure equivalent training experiences in the two groups.

#### Mother-child pairs

Children aged 2 months to five years who were sick and brought to the clinic by their mothers were eligible to participate in the evaluation study. Children attending with injuries or for well child visits were excluded. Children attending without the mother or where the mother was aged below 18 years were also excluded because of difficulties obtaining informed consent from non-maternal carers or from mothers aged below 18 years. Mothers of all eligible children were approached to participate.

### Sample size

An initial sample size of 125 was calculated based on detecting a 20% difference in the primary outcome, which was the proportion of children who received all treatments indicated with a probability of 95%, 80% power and a baseline estimate of 70%. We therefore aimed to detect an absolute increase in children receiving all treatments from 70% -84%. An ICC of 0.015 was assumed resulting in a design effect of 1.4. Therefore, required sample size was 180 (or 90 per group). Six children from 15 clinics were selected in each arm of the study.

### Data collection

At the start of the IMCI update training IMCI trained nurses in both groups completed a self-administered questionnaire to determine basic demographic data and participants’ confidence regarding computer use. After the IMCI update training an IMCI knowledge questionnaire was administered to all participants to determine their IMCI knowledge before they returned to their work station.

Data about the clinical performance of participants were collected using a quantitative survey conducted among mother-child pairs in all participating clinics, which began four months after the start of eIMCI implementation. Data were collected by two field teams, each comprising an IMCI expert practitioner and a field worker who assisted with organization and logistics. IMCI experts were professional nurses, each with more than 10 years of IMCI experience. After the mother exited the consulting room the IMCI expert repeated the IMCI assessment for each participating child to provide the gold standard against which nurse participant’s practice was assessed. Field teams received two weeks training including a detailed review and practice of the IMCI guidelines, as well as training in data collection processes according to standard operating procedures for the study. All tools were piloted and adapted during training.

Data were collected for one week in each participating clinic or until a minimum of 7 children were enrolled from each clinic. All mother- child pairs attending the clinic were assessed for eligibility while waiting in the queue, and all eligible mothers were approached to participate. All children aged between 2 months and five years were given an armband while waiting. This was to ensure that the IMCI practitioners could not identify study participants from among other clinic attendees. Eligible children were differentiated from other children in the queue by affixing an inconspicuous mark on the armband for tracking purposes. When an eligible child came to the front of the queue field workers noted the time of entry and exit from the consulting room.

Quantitative data were collected by the IMCI expert using a series of structured data collection tools as follows: 1) a repeat IMCI assessment tool to record the correct assessment and classification for each child, 2) a review of the child’s records to record all classifications written by the IMCI practitioner, 3) medication provided to the child was reviewed and recorded, 4) the child’s clinical records were scanned to provide a clear record of IMCI practitioners findings. Data were collected in the local language (isiZulu) or in English according to the participant’s preference. If a child had a severe classification data were collected once all pre-referral treatments were complete and the child was waiting for the ambulance.

### Analysis

Descriptive statistics were used to summarise the data. Demographic data were presented as frequencies. Knowledge data were scored to provide an IMCI knowledge score for each participant. The times of entry and exit to the consulting room were used to determine the duration of consultations in the eIMCI and pIMCI groups.

Classifications made by the IMCI experts were considered as being correct for the purposes of the analysis. Classifications made by eIMCI and pIMCI practitioners were recorded during data collection and were validated during data analysis using the scanned records. Classifications made by study participants were compared with the classifications of the same child by the IMCI expert to determine the proportion of children who received a correct classification for each condition. The proportion of children classified correctly was compared between eIMCI and pIMCI practitioners to determine the performance of practitioners in the two groups.

Correct medications for each participating child were determined after completion of data collection. Two IMCI experts independently identified correct medications for each child based on the findings of the IMCI expert. Medications identified by the two IMCI experts for each enrolled child were compared and any differences discussed and resolved to obtain a final list of correct medications. The proportion of children who received correct medications for each condition was determined by comparing the medications given to the child to the correct medications as determined by the IMCI experts.

Categorical data such as correct identification of symptoms and prescribing practices were compared using Chi Square tests. Design based p-values were reported after adjusting for the effect of the clustering of patients within a clinic. Numeric data was summarized using Medians, IQR with minimum were used to summarise numeric data and Mann Whitney tests used to compare the two groups. Svy commands in STATA v17 was used for statistical analysis.

## Results

30 IMCI trained health workers were enrolled in the study (15 per group) and received eIMCI/pIMCI training during October 2020. On completion of the training IMCI knowledge was assessed among all participants and found to be similar in the intervention group (median 59.6%; IQR 40-89) and control group (63.8%; IQR 55-76) with no significant difference in IMCI knowledge between the groups (*p*=0.76). All participants received at least 2 mentoring visits between October and December 2020. Demographic characteristics of enrolled IMCI practitioners and their computer self-efficacy are shown in Table 1.

**Table 1:** Demographic characteristics and self-reported computer skills among IMCI practitioners

***N*=30**	**pIMCI group** **n (%)**	**eIMCI group** **n (%)**	**All** **n (%)**
**Age**	Mean 38 (27-52) SD 8.7	Mean 43 (26-59) SD10.5	Mean 40 (26-59) SD 9.8)
**Gender**			
Male	3	2	5
Female	12	13	25
**Race**			
African	15	14	29
Indian	0	1	1
**How long ago were you trained in IMCI**			
Less than 1 year ago	2	0	2
1 to < 3 years ago	6	5	11
3 to < 6 years ago	1	3	4
More than 6 years ago	4	6	10
Cannot remember	2	1	3
**How confident do you feel about using a computer?**			
Not very confident	5	6	11
Somewhat confident	5	5	10
Very confident	0	3	3
Missing data	5	1	6
**Ever attended computer training**	1	4	5
**Owns their own computer**	2	3	5

Survey data were collected in participating clinics between February and June 2021. IMCI was used during all assessed consultations in both groups: all eIMCI practitioners used eIMCI and all enrolled children had an eIMCI printout; pIMCI practitioners all completed a paper IMCI recording form, except in one clinic where these are unavailable. A total of 291 children were enrolled in the study comprising 152 in the eIMCI group and 139 in the pIMCI group. The mean number of enrolled children was 9.7 per clinic (range 7-12). Mean ages of enrolled children in months were similar in both groups (20.1 (SD 14.7) vs 20.1 (SD 14.9): *p*= 0.9). The duration of the consultation was recorded and results are shown in Table 2.

**Table 2:** Duration of eIMCI and pIMCI consultations

	**N**	**Median time/ minutes**	**IQR/minutes**	**Min, max (minutes)**	***P***
**pIMCI**	139	25	17-32	6, 74	0.02
**eIMCI**	150^a^	28	20-35	4, 74	
**Total**	289	26	18-32	4, 74	

^a^missing data for two participants

### Performance of IMCI practitioners compared to gold standard IMCI assessments

The classifications made by the IMCI practitioners in both intervention and control groups were compared to the assessment by the IMCI expert. To determine inter-rater reliability the two IMCI experts assessed the same 12 children independently. They agreed on 181/192 classifications giving a 94.3% equivalence between the two experts. The numbers of children with severe classifications requiring urgent referral were few; 5 severe classifications in eIMCI group and 4 in the pIMCI group. Among 5 children with severe classification seen by eIMCI participants, 3 were correctly identified, and among 4 children with severe classification in the pIMCI group, 2 were correctly identified.

Table 3 shows the proportion of children with each main symptom (cough or difficult breathing, diarrhoea, fever, ear problem) or screening condition (nutrition, HIV, TB) correctly classified by eIMCI practitioners and pIMCI practitioners.

**Table 3:** Performance of eIMCI and pIMCI practitioners in the classification of sick children

	**eIMCI** *N*=152		**pIMCI** *N*=139		**Adjusted. *p* value**
**Symptom**	Symptom present	Correctly classified n (%)	Symptom present	Correctly classified n (%)	
Cough or difficult breathing	76	57 (75.0)	73	65 (89.0)	0.06
Dehydration	25	17 (68.0)	12	11 (91.7)	0.16
Fever	44	16 (36.4)	43	29 (67.4)	0.01
Ear infection	5	3 (60.0)	10	7 (70.0)	0.6
Children with all presenting symptoms correctly classified	109	59 (54.1)	105	61(58.1)	0.67
**Screening conditions**					
Malnutrition	152	115 (75.7)	139	87 (62.6)	0.07
Anaemia	152	149 (98.0)	139	83 (59.7)	<0.001
HIV	152	106 (69.7)	139	50 (36.0)	<0.001
TB	152	124 (81.6)	139	100 (71.9)	0.17
Children with all screening conditions correctly classified	152	79 (52.0)	139	25 (18.0)	<0.01

Table 3 shows that, despite using the eCDSS, many eIMCI practitioners failed to make correct classifications for presenting symptoms, and pIMCI practitioners performed consistently better for all main symptoms. The most commonly reported symptoms were cough or difficult breathing and fever, and for these two symptoms the most frequent reason for an incorrect classification was because the eIMCI practitioner failed to enter the symptom into eIMCI and therefore no assessment was done. For example, among 76 children with cough or difficult breathing in the eIMCI group, 19 children received an incorrect classification comprising 12 children where the symptom was omitted and 7 who were classified incorrectly. Similarly, among 44 children with fever in the eIMCI group 28 children were classified incorrectly, and 27 were not classified because the practitioner omitted to record the symptom on eIMCI. Among all symptom classifications eIMCI practitioners were more likely to omit a presenting symptom compared to pIMCI practitioners (45/150; 30% vs 19/138; 13.7%: *p*=0.001).

In contrast, Table 3 also shows that eIMCI practitioners were more likely to make correct classifications for screening symptoms compared to pIMCI practitioners who often omitted these classifications. To determine whether this resulted in more children at-risk of malnutrition, TB and HIV being identified, the proportion of children who screened positive that were correctly identified by eIMCI and pIMCI practitioners is shown in Table 4. Table 4 shows that the proportion of children correctly identified as having malnutrition or being at risk of malnutrition, TB or HIV was low in both eIMCI and pIMCI groups (19/59; 32.2% vs 8/43; 18.6%, *p*= 0.12).

**Table 4:** Proportion of children screening positive identified by eIMCI and pIMCI practitioners

	**eIMCI**		**pIMCI**	
	**Number screened positive (gold standard)**	**Number identified by eIMCI practitioner**	**Number screened positive (gold standard)**	**Number identified by pIMCI practitioner**
**Anaemia**	1	0	1	0
**Severe acute malnutrition +/- medical complications**	2	2	2	1
**Moderate acute malnutrition**	7	6	6	0
**Not growing well**	16	4	13	4
**HIV infection**	1	1	1	1
**Symptomatic HIV infection**	3	0	3	0
**High risk of TB**	2	1	1	0
**Risk of TB**	27	5	16	2
**All positive screening conditions**	59	19	43	8

### Performance of IMCI practitioners in providing medication to sick children

Medications provided to sick children were compared to the medication identified by the IMCI experts to determine whether children received essential treatments and whether there was over-prescription of medications that were not indicated. In addition, coverage of preventive treatments (Vitamin A, mebendazole, and immunization) were compared based on whether these preventive treatments were up to date according to the child’s patient held record (Road-to-Health-Book). Table 5 shows that eIMCI practitioners were less likely to prescribe all medications that were indicated according to IMCI guidelines, and were more likely to overprescribe by providing medications not indicated by IMCI.

**Table 5:** Prescribing practices of eIMCI and pIMCI practitioners

	**eIMCI**	**pIMCI**	**ALL**	**Adjusted *p*-value**
	*N*=152 n (%)	*N*= 139 N (%)	*N*= 291	
All curative meds given today (cough, diarrhoea, fever, ear infection)	124 (81.6)	126 (90.6)	250 (85.9)	0.0695
HIV treatment correct	5/30 (16.7)	4/33 (12.1)	9/63 (14.3)	0.66
TB treatment correct	0	0	0	n/a
RTHB REVIEW				
Vit A up to date	128 (84.2)	130 (93.5)	258 (88.7)	0.03
Deworming up to date	124 (81.6)	127 (91.4)	251 (86.2)	0.15
Immunisation up to date	145 (95.4)	134 (96.4)	279 (95.9)	0.65
**OVERPRESCRIPTION**				
Any over-prescription (includes any medication not indicated by IMCI)	48 (31.6)	20 (14.4)	68 (23.4)	0.004
Unnecessary antibiotic given	16 (10.5)	7 (5.0)	23 (7.9)	0.12
Multivitamins given when not indicated	27 (17.8)	8 (5.8)	35 (12.0)	0.003

## Discussion

The study shows that implementing an eCDSS to support IMCI implementation in PHC clinics in KZN, South Africa, failed to improve clinical care for sick children aged 2 months to five years. The motivation for implementing eIMCI in our setting was to improve adherence to the guidelines and improve prescribing practices [1]. However, our study showed that these aims were not achieved. eIMCI practitioners reported poor computer skills, missed out components of the algorithm, failed to identify most children at-risk of screening conditions, and were more likely than pIMCI practitioners to prescribe an unnecessary antibiotic. A process evaluation conducted alongside the cluster RCT and presented elsewhere showed poor eIMCI uptake during the eIMCI implementation period [28]. These findings contrast with the findings of studies of other electronic IMCI support systems, where the eCDSS resulted in a more comprehensive assessment and more rational and consistent prescribing [4]. However, in other studies eIMCI practitioners were directly observed, which could have resulted in better uptake of the intervention [21, 22]

The aim of eIMCI implementation was to standardize IMCI assessments, and to ensure that children received comprehensive care at every visit. Management of sick children is complex and eIMCI provided a stricter framework for IMCI implementation that was less reliant on the ability of individual IMCI practitioners to classify correctly and identify all required treatments using multiple algorithms [20]. As eIMCI practitioners entered the clinical history and findings into the computer when prompted, eIMCI generated classifications, treatments and counselling messages for each child. However, our study showed that eIMCI practitioners were more likely than pIMCI practitioners to omit presenting symptoms, and since a symptom cannot be omitted once entered, this suggests that eIMCI practitioners failed to record that these symptoms were present. This may have been a result of poor computer skills leading to difficulties and errors in navigating eIMCI, in particular moving backwards to include symptoms mentioned by mothers later in the consultation. High workloads and time pressure are likely to have contributed to practitioners omitting symptoms.

The assumption that making all components of the eIMCI assessment mandatory would result in more comprehensive IMCI implementation, failed to consider or address underlying reasons for poor IMCI implementation. Our findings suggest that practitioners skip over aspects of the algorithm even when these are compulsory and fail to identify signs of underlying conditions even when the assessment of these is mandatory. We suggest that the reasons for poor eIMCI implementation may be the same as for poor IMCI implementation generally, which include inadequate knowledge and training, lack of confidence in the guidelines, overwork, lack of motivation and time constraints[17]. Undertaking a comprehensive assessment is time consuming in busy clinics, and using a computer-based system to force practitioners to work through all components of the algorithm is unlikely to lead to effective implementation unless practitioners are convinced of the value of these assessments. Studies have found that to be successful it is important that an eCDSS does not disrupt the work flow or inconvenience practitioners as this will discourage its use [1], and more than half ultimately fail [29]. eIMCI consultations took significantly longer than pIMCI consultations, and it is likely that having to do a comprehensive assessment contributed to this. Any initiative that is perceived as adding to the workload is unlikely to be accepted.

The proportion of children receiving correct classifications for screening symptoms (malnutrition, HIV and TB) was higher in the eIMCI group, where completion of screening assessments was mandatory, suggesting a more comprehensive assessment of screening conditions by eIMCI practitioners compared to pIMCI practitioners. However, identification of screening conditions was poor in both groups, and the improvement was mainly due many children receiving correct negative classifications. However, the increased identification of positive screening conditions, particularly acute malnutrition, was a clinically significant improvement and was likely to be because weight-for-height Z-scores are automatically calculated by eIMCI, rather than relying on individual pIMCI practitioner’s calculations. Only a small proportion of children screen positive but it is important that they are identified to prevent life-threatening illness. Possible explanations for poor performance include that eIMCI practitioners lacked the skills to identify signs of anaemia, HIV or TB, or that they skipped through these components of the algorithm by guessing the most likely answers to save time. It is notable that in South Africa the TB and HIV screening algorithms are local adaptations, and are lengthy, complex, and time-consuming, which may have contributed to a reluctance to implement these algorithms.

Another key aim of an eCDSS is to improve rational prescribing [4], but our study showed no improvement in correct prescribing among eIMCI participants. On the contrary, eIMCI practitioners were more likely to give unnecessary medication. One in 10 children managed by eIMCI practitioners received an antibiotic not indicated by IMCI, more than double the proportion in the pIMCI group. This is in contrast to other studies which have shown substantial improvement in prescribing practices [21, 30]. However, several studies have also highlighted conflict and uncertainly when health workers disagreed with the eCDSS, and in some cases users were unhappy that the tool prevented them from making decisions based on their own clinical acumen [5]. This highlights the complexity of decision-making around clinical practice and the importance of building confidence in the new system. It is well established that providing guidelines alone is insufficient to change clinical practice, and there is no reason why this should be different just because the guidelines are electronic. More research is required to understand the underlying reasons for deviating from recommended prescribing practices.

Another important barrier to eIMCI implementation was the lack of computer skills among eIMCI practitioners, which was likely to have created multiple barriers and disincentives to eIMCI implementation, including contributing to the increase in consultation time. Currently computers in clinics are only used for administrative purposes and eIMCI nurses had little or no experience with computers either at home or in the workplace, and were unfamiliar with the concept of using a computer during the consultation [28]. Other studies across Africa show that heath workers frequently have poor computer knowledge and skills, as well as poor access to computers [7], so it is important to pilot new eCDSSs carefully to ensure that computer skills are not a barrier to implementation [31]. In particular, it is likely that the decision to use desktop computers rather than a tablet computer with a touch screen, which would have been more user friendly and portable, had an adverse effect on implementation. Using a desktop computer prevented eIMCI practitioners from using eIMCI when they were deployed in other areas of the clinic.

These findings should be considered in the light of the potential broader benefits of using eCDSSs in the future, including that guidelines can be quickly and easily updated to ensure that they are always current without the need for re-training, provided the changes are not substantial and do not require additional clinical skills. Electronic guidelines have potential to provide a health information system to record clinic attendances for sick children, thereby substantially reducing or eliminating the need to collect data. eCDSSs can be an important tool for facilitating task shifting to lower cadres of health worker, for example eIMCI could be adapted to provide guidelines for Community Health Workers to assess sick children in the household. These benefits will increase as more electronic or e-Health initiatives are adopted, and the infrastructure and support costs are shared between a variety of initiatives. However, careful attention needs to be paid to the barriers to implementation including aligning the eCDSS with existing workflow, supporting computer skills, and building trust and confidence in the expert system.

### Strengths and weaknesses

This study employed a strong methodology. However, it is likely that the participants performance was affected by the presence of the research team in the clinic, although researchers were not present in the consulting room, particularly given the high uptake of eIMCI during the data collection compared to during the implementation [28] . A limitation of the repeat assessment by the IMCI expert was the possibility that the clinical condition of the child could have changed between the two assessments. To minimize this the reassessment was done as soon as possible after the completion of the consultation. Although this possibility cannot be excluded it should apply equally to the intervention and control groups. Another weakness was that the sample size was inadequate to evaluate IMCI practitioners’ performance in assessing those conditions that were rarely seen, for example severe classifications, or positive screening conditions.

## Conclusions

While these findings are discouraging, given the huge potential benefits of eCDSSs it is possible that eIMCI may be able to support clinical decision-making in the future, if challenges are explored and addressed ahead of implementation. Our findings highlight the need for careful piloting and formative evaluations of implementation to understand challenges and constraints, both for practitioners and the wider health system, ahead of a larger scale roll out of e-health interventions. In addition, our findings highlight the importance of undertaking robust evaluations of even the most promising new initiatives, not only to determine their effectiveness but also to explore reasons for poor performance where relevant.

## Data Availability

The datasets used are not currently available because further analysis is underway but are available from the corresponding author upon reasonable request.

## Abbreviations

eIMCI: Electronic Integrated Management of Childhood Illness
eCDSS: Electronic clinical decision support system
FGD: Focus group discussion
IDI: In-depth interview
IMCI: Integrated Management of Childhood Illness
LMIC: low- and middle-income countries
pIMCI: Paper-based IMCI
PDA: Personal digital assistant
PMTCT: Prevention of mother-to-child transmission of HIV
PHC: Primary health care
UNICEF: United Nations Children’s Fund
WHO: World Health Organization
